# CT-perfusion stroke imaging: a threshold free probabilistic approach to predict infarct volume compared to traditional ischemic thresholds

**DOI:** 10.1038/s41598-017-06882-w

**Published:** 2017-07-27

**Authors:** Fabian Flottmann, Gabriel Broocks, Tobias Djamsched Faizy, Marielle Ernst, Nils Daniel Forkert, Malte Grosser, Götz Thomalla, Susanne Siemonsen, Jens Fiehler, André Kemmling

**Affiliations:** 1 Department of Diagnostic and Interventional Neuroradiology, University Medical Center Hamburg-Eppendorf, Hamburg, Germany; 2 Department of Radiology and Hotchkiss Brain Institute, University of Calgary, Calgary, Canada; 3 Department of Neurology, University Medical Center Hamburg-Eppendorf, Hamburg, Germany; 4 Institute of Neuroradiology, University Medical Center Schleswig Holstein, Lübeck, Germany

## Abstract

The aim was to evaluate a novel method of threshold-free prediction of brain infarct from computed tomography perfusion (CTP) imaging in comparison to conventional ischemic thresholds. In a multicenter cohort of 161 patients with acute large vessel occlusion who received endovascular therapy, brain infarction was predicted by CTP using (1) optimized parameter cut-off values determined by ROC curve analysis and (2) probabilistic logistic regression threshold-free analysis. Predicted infarct volumes and prediction errors based on four perfusion parameter maps were compared against observed infarcts. In 93 patients with successful recanalization, the mean observed infarct volume was 35.7 ± 61.9 ml (the reference for core infarct not savable by reperfusion). Optimal parameter thresholds predicted mean infarct volumes between 53.2 ± 44.4 and 125.0 ± 95.4 ml whereas threshold-free analysis predicted mean volumes between 35.9 ± 28.5 and 36.1 ± 29.0 ml. In 68 patients with persistent occlusion, the mean observed infarct volume was 113.4 ± 138.3 ml (the reference to define penumbral infarct savable by reperfusion). Predicted mean infarct volumes by parameter thresholds ranged from 91.4 ± 81.5 to 163.8 ± 135.7 ml, by threshold-free analysis from 113.2 ± 89.9 to 113.5 ± 89.0 ml. Threshold-free prediction of infarct volumes had a higher precision and lower patient-specific prediction error than conventional thresholding. Penumbra to core lesion mismatch estimate may therefore benefit from threshold-free CTP analysis.

## Introduction

In acute stroke, CTP imaging is used to identify patients eligible for treatment^1–3^. CTP imaging allows differentiation of infarct core (irreversibly damaged tissue regardless of reperfusion) and penumbra (critically hypoperfused tissue destined to infarct in case of persistent occlusion)^4, 5^. A correct volumetric prediction of lesion mismatch between core and penumbra can identify patients who will benefit from recanalization^6^.

Recent studies have used clearly defined inclusion criteria based on volumetric core/penumbra lesion mismatch ratios^3, 7, 8^. A small irreversible lesion core volume in relation to a large potentially salvageable penumbral lesion volume indicates high probability of benefit after recanalization therapy. Thus, improving the precision of volumetric prediction of salvageable tissue will likely improve the selection of patients that may ultimately benefit from recanalization while avoiding potentially harmful futile treatment of patients unlikely to benefit from this procedure.

Traditionally, thresholds have been applied to CT perfusion parameter maps to categorize voxels into “infarction” or “no infarction” depending on recanalization or reperfusion status, and optimal thresholds have been determined to identify the core lesion and penumbra accordingly^9–12^. To find the optimal perfusion parameter threshold that quantifies ischemic core and penumbra, tissue infarct lesions in patients after successful recanalization and permanent occlusion have been used as reference, respectively^10, 12^. Thus, an ideal volumetric core/penumbra lesion assessment correctly predicts the final infarct volume in case of successful recanalization (i.e. the reference for the core lesion), as well as the final volume of infarct if no recanalization is achieved. The difference between these two predicted volumes corresponds to the tissue potentially rescuable by recanalization therapy (the penumbra, or tissue-at-risk).

A threshold for voxel classification into core and penumbra seems pathophysiologically appealing and suggests that there may be a physiological “switch-like” cut-off value above or below which ischemic brain tissue evolves into infarction. However, this dogmatic approach seems degraded by oversimplification and the technical approach of thresholding has severe limitations. Dichotomizing a perfusion parameter image by a predefined cut-off value leads to information loss, as the continuous perfusion parameter is transformed into a binary variable (infarction/no infarction) that cannot differentiate between different risk levels of infarct. This information loss may lead to imprecise estimates of infarct volume by conventional thresholds. Furthermore, the operational definition of the optimal threshold after ROC-curve analysis varies.

**Figure 1:**
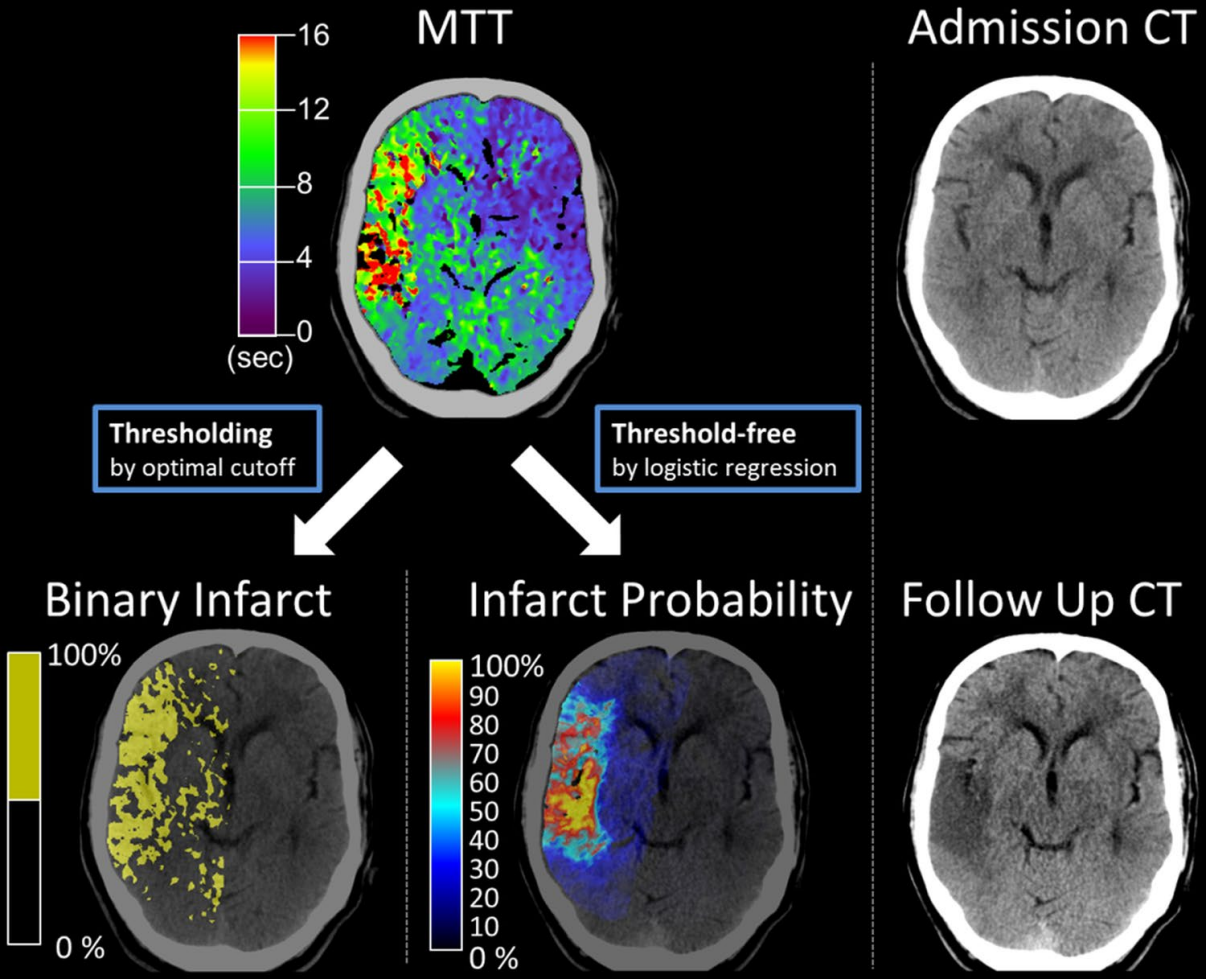
161 acute stroke CTP maps were processed using traditional thresholding and the probability threshold-free method. Traditional thresholding generates a binary map of predicted infarction vs. no infarction. The threshold-free method uses logistic regression to generate a continuous voxel-wise map of infarct probability, and the sum of all infarct probabilities equals the predicted infarct volume. The infarct volumes predicted by both methods were compared to the real infarct volumes observed on follow up CT.

In a previous study, a threshold-free approach has been used in a multivariate CTP model in order to dynamically predict infarction depending on multiple parameters including time to recanalization in acute stroke patients^13^. It remains unclear, however, how this threshold-free approach performs in traditional univariate prediction of infarct volumes using single perfusion parameter maps in comparison to conventional thresholding. To address these questions, we analyzed data of a previously published multicenter patient cohort. We predicted infarct volumes (1) threshold-based by calculating and applying optimal cut-off values for CTP maps as previously described^10^, and (2) by applying a univariate threshold-free approach. The univariate threshold-free approach converts a continuous perfusion parameter map via logistic regression into statistical infarct probabilities and directly uses these probabilities to predict infarct volumes without applying a threshold (Fig. 1). The aim of the study was to evaluate the precision of volumetric tissue outcome prediction from separate univariate analysis of four perfusion parameter maps [cerebral blood volume (CBV), cerebral blood flow (CBF), mean transit time (MTT), and time to drain (TTD)] using (1) the traditional method of thresholding compared to (2) the alternative threshold-free approach.

## Materials and Methods

### Patients

For this study, anonymized data from prospectively collected stroke registries (9–2008 to 12–2012) from four academic primary stroke centres were pooled as previously described^13^. The study was approved by the ethical review board (Ethik-Kommission der Ärztekammer Hamburg) and was conducted according to the Helsinki guidelines for human experiments.

Inclusion criteria to this study were as follows:

(i) First incident of acute ischemic stroke with known time of clinical onset,
(ii) National Institutes of Health Stroke Scale (NIHSS) score above 3,
(iii) Admission imaging consisting of a multimodal computed tomography (CT) imaging protocol including CT-angiography (CTA) and CTP and follow-up imaging at 48 hours to 7 days for final infarct segmentation,
(iv) Proximal (M1 segment) occlusion of the middle cerebral artery (MCA) or intracranial carotid artery (ICA) verified by CTA,
(v) Endovascular treatment with known recanalization time and degree of recanalization, and
(vi) Time from clinical onset to admission imaging not exceeding 7 hours.

Patients with evidence of significant cerebral haemorrhage leading to death or clinical deterioration (>4 NIHSS points) were excluded^14^.

The Thrombolysis in Cerebral Infarction (TICI) score was determined in digital subtraction angiography (DSA) acquired during endovascular treatment. Patients were divided into two groups according to TICI score: successful recanalization (TICI 2b-3) and unsuccessful recanalization with persistent occlusion (TICI 0-2a)^15^.

### Imaging

The imaging protocol at admission included a standard non-contrast CT, CTA, and spatio-temporal (4D) CTP, which were always acquired in this order on 64 or 128 dual slice CT scanners (Siemens Definition AS+; Siemens Definition Flash; Philips Brilliance 64). A follow-up standard non-contrast CT was acquired between 48 hours and 7 days after admission.

The imaging parameters used were as follows: CT: 120 kV, 280–320 mA, with 5.0 mm slice reconstruction. CTP: 80 kV, 200–250 mA, with 5 mm slice reconstruction (max. 10 mm), slice sampling rate of 1.50 s (min. 1.33 s), scan time 45 s (max. 60 s), biphasic injection with 30 ml (max. 40 ml) of highly iodinated contrast medium with 350 mg iodine/ml (max. 400 mg/ml) injected with at least 4 ml/s (max. 6 ml/s) followed by 30 ml saline solution bolus. CT and CTP images were interpolated to an in-slice resolution of 0.37 mm with 5.0 mm slice thickness. CTA: 100–120 kV, 260–300 mA, with 1.0 mm slice reconstruction, 5 mm maximum intensity projection (MIP) reconstruction with 1 mm increment.

### Perfusion CT data post processing

Whole brain perfusion maps of CBV, CBF, MTT, and TTD were computed based on the CTP datasets. CTP data were processed on a dedicated workstation for perfusion analysis (Syngo mmwp VE52A; Siemens Healthcare, Forchheim, Germany), including motion correction, low band temporal noise removal, and automatic exclusion of non-parenchymal voxels. Least mean squares deconvolution was used to calculate perfusion parameter maps^16^. The reference arterial input function (AIF) required for this was automatically measured in the middle and anterior cerebral artery, and the venous outflow and maximum enhancement was quantified in the superior sagittal sinus for correction of the AIF. The automatically selected arterial and venous reference voxels were visually checked in all cases. CTP datasets with severe motion artifacts, incomplete or flawed attenuation time curves, or incomplete MCA territory coverage were excluded. All perfusion maps were interpolated to 5 mm slice thickness using nearest neighbor interpolation to ensure that image grey values remain unaltered. In MTT and TTD parameter maps, brain voxels with very low cerebral blood volume below attenuation levels to detect timing of contrast bolus were automatically excluded from the analysis.

### Perfusion parameters as predictor variables

Quantitative perfusion maps acquired at the time of patient admission served as predictor variables of final infarct volume. Predicted infarct volumes were calculated in each patient in each group (successful recanalization/persistent occlusion) by univariate analysis of each perfusion parameter map separately (CBF/CBV/MTT/TTD) using each respective method (infarct prediction by optimal perfusion threshold vs. infarct prediction by threshold-free analysis) as explained below.

### Response variable

Final tissue outcome was classified voxel-wise as a binary response variable (1 = infarct, 0 = no infarct). For this purpose, infarct lesions on follow-up imaging CT were segmented manually by an experienced observer. Each follow-up image was then registered to the baseline time-average image of the CTP dataset using affine transformation (Analyze 11.0, AnalyzeDirect).

### Generalized linear model

We adapted a previously described generalized linear model (GLM)^13, 17^ to calculate maps of infarct probability from perfusion parameter maps. The GLM was trained based on real final tissue outcome as response variable. Because the observed response variable was binary (follow-up infarction/no infarction), a logit function was used as link function for training the GLM. The resulting logistic regression model assigns a conditional infarct probability ${\pi }(x)$ between 0 and 1 to each voxel depending on its perfusion parameter value as the univariate independent predictor variable. Logistic regression models were trained for CBV, CBF, MTT, and TTD parameter maps separately to convert each perfusion parameter map to a map of infarct probability in each group of patients (successful recanalization/persistent occlusion).

### Infarct volume prediction

For the conventional threshold-based method, optimal thresholds were determined for each perfusion parameter by ROC curve analysis with respect to final tissue outcome (see statistical analysis below). The optimal threshold was then applied to the corresponding perfusion map for each patient. All voxels with values higher than (in case of MTT and TTD) or less than (in case of CBF and CBV) the corresponding parameter threshold were classified as infarct voxels (value = 1). All remaining voxels were classified as non-infarct voxels (value = 0). The expected infarct volume was calculated as the sum of infarct voxels (this is equivalent to the product between the total number of sampled brain voxels and the average infarct value per voxel).

For the threshold-free approach, voxel-wise perfusion values were converted to voxel-wise infarct probability by GLM based logistic regression analysis as explained above. The expected infarct volume was then statistically calculated as the cumulative sum of infarct probabilities across all voxels (this is equivalent to the product between the total number of sampled brain voxels and the average infarct probability per voxel)^18^. The total infarct volume in millilitres was thus computed by multiplying the total number of sampled voxels by the average voxel infarct probability and voxel volume.

For both methods, only voxels within the ischemic hemisphere (opposed to the entire perfusion map) were used as the training data set in order to avoid true-negatives in the healthy hemisphere where infarct prediction is meaningless and may exaggerate the specificity of thresholds and area under the receiver operating characteristics curve (AUC) after ROC curve analysis^4^.

### Statistical analysis

The perfusion parameter maps and the response variable map (binary map of final infarction) of both patient groups (successful and failed recanalization) were converted to a voxel-wise data matrix (Matlab R2014a). The sample space of each patient consisted of all parenchymal voxels in the affected brain hemisphere. The voxel-wise data matrix was exported to R (Version 3.02, EPI package) to (i) derive the optimal threshold values (i.e. optimal cut-off values discriminating infarct from non-infarct) for each parameter map by ROC curve analysis using the Youden-Index^19^ and to (ii) calculate the GLM coefficients using the iterative maximum-likelihood estimation method. Coefficients were tested for robustness with leave-one-patient-out cross validation^20^.

The root mean square error (RMSE) was used to determine the group-wise accuracy of predicted infarct volumes vs. observed infarct volumes. The RMSE quantifies the mean volumetric prediction error across all patients (the ideal RMSE would be 0 ml, *i*.*e*. the predicted and real infarct outcome match exactly for every patient) and is defined by:

$$(5)\,{\rm{RMSE}}=\sqrt{{\sum }_{1}^{{\rm{N}}}{({{\rm{V}}}_{{\rm{p}}}-{{\rm{V}}}_{{\rm{o}}})}^{2}/{\rm{N}}}$$

V_p_ denotes the predicted infarct volume, V_O_ the observed infarct volume and N the number of patients. The 95 percent confidence interval (CI) of the RMSE was estimated by bootstrapping 100000 random samples. Group differences were statistically analyzed with a randomization test (100000 random samples), which is independent of the distribution of the underlying data^21^.

The two patient groups (successful vs. non-successful recanalization) were compared with an unpaired t-test (normally distributed values) and Mann-Whitney U rank-sum test (non-normally distributed values) for quantitative continuous or discrete variables. Fischer’s exact test was used for qualitative categorical variables. Correlations were analyzed with Pearson correlation coefficient. The level of significance was defined with two-sided α ≤ 0.05. Continuous variables are shown as mean and standard deviation (SD). Discrete variables are reported as counts or percentages.

## Results

A multicenter dataset of 161 endovascular treated patients with acute proximal MCA occlusion was analyzed (Table 1). Of these, 93 patients (58%) were successfully recanalized (TICI 2b-3), whereas 68 patients (32%) were not successfully recanalized and showed a persistent occlusion (TICI 0-2a), as previously published^13^.

**Table 1:** Patient characteristic table, as previously published^13^.

Baseline characteristics	All Patients	TICI 2b-3	TICI 0-2a	*P*
Subjects, n (%)	161 (100.0)	93 (57.8)	68 (42.2)	
Age, years, mean (SD)	69.0 (14.3)	68.5 (14.4)	69.6 (14.3)	0.64
Male sex, n (%)	72 (44.7)	42 (45.6)	30 (44.1)	1.00
Admission NIHSS, median (IQR)	16 (12–18)	15 (11–18)	16 (13–19)	0.03
Discharge mRS, median (IQR)	4 (1–5)	2 (0–4)	4 (1–5)	<0.01
Vessel occlusion				
MCA main stem, n (%)	138 (86)	86 (92.5)	52 (76.5)	<0.01
Carotid-T, n (%)	23 (14)	7 (7.5)	16 (23.5)	<0.01
Etiology				
Atherothrombotic, n (%)	16 (9.9)	8 (8.6)	8 (11.8)	0.60
Cardioembolic, n (%)	103 (64.0)	64 (68.8)	39 (57.4)	0.13
Undetermined etiology, n (%)	36 (22.4)	18 (19.4)	18 (26.5)	0.13
Other etiology, n (%)	6 (3.7)	3 (3.2)	3 (4.4)	0.70
IV Bridging, n (%)	126 (78.0)	76 (81.2)	50 (73.5)	0.14
IA Treatment, n (%)	161 (100.0)			
Mechanical only, n (%)	85 (52.8)	52 (55.9)	33 (48.5)	0.42
Thrombolysis and mechanical, n (%)	32 (19.9)	17 (18.3)	15 (22.1)	0.56
Thrombolysis only, n (%)	44 (27.3)	24 (25.8)	20 (29.4)	0.72
Time from onset to				
Admission imaging, h, mean (SD)	2.3 (1.5)	2.4 (1.5)	2.2 (1.5)	0.49
Recanalization, h, mean (SD)	5.0 (1.7)	5.0 (1.6)	5.0 (1.8)	0.72
Final tissue outcome				
Infarct, ml, median (IQR)	41.4 (11.5–109.7)	31.6 (12.0–92.5)	147.0 (59.1–228.3)	<0.001
Infarct, % hemisphere, median (IQR)	14.5 (3.8–34.3)	6.4 (2.4–18.7)	29.7 (11.9–46.1)	<0.001

### Threshold method: AUC and optimal cutoff values of perfusion parameters

Infarct prediction was performed for the two patient groups: (A) patients with successful recanalization and (B) patients with persistent occlusion. For all patients in each group, four separate perfusion parameter maps (CBF, CBV, MTT, TTD) aligned with final infarct lesion maps were used separately to determine ROC curves and calculate AUC values (Table 2).

**Table 2:** Area under the receiver operating characteristics curve (AUC) values to determine infarction and optimal cutoff values (thresholds) of perfusion parameters.

	AUC values		Optimal cutoff values	
	TICI 2b-3 (n = 93)	TICI 0-2a (n = 68)	TICI 2b-3 (n = 93)	TICI 0-2a (n = 68)
CBF	0.671	0.673	33 ml × 100 g^−1^ × min^−1^	34 ml × 100 g^−1^ × min^−1^
CBV	0.628	0.593	2.4 ml × 100 g^−1^	2.4 ml × 100 g^−1^
MTT	0.535	0.586	10.0 s	9.0 s
TTD	0.614	0.673	9.7 s	8.2 s

AUC values ranged from 0.535 (MTT in TICI 2b-3) to 0.673 (CBF and TTD in TICI 0-2a). The power of perfusion parameter maps to predict infarct (as designated by AUCs) was different in patients with successful recanalization and patients with persistent occlusion. The perfusion maps with the highest AUC were CBF for patients with successful recanalization (AUC: 0.671) and both CBF and TTD for patients with persistent occlusion (AUC: 0.673).

To define the optimal cutoff values for conventional threshold-based infarct prediction, the Youden indices (parameter cutoff value where specificity and sensitivity are maximized) were used from the ROC curves. MTT and TTD perfusion parameters showed different optimal cutoff values in patients with successful recanalization vs. patients with persistent occlusion while CBV and CBF were rather similar (Table 2). The optimal cutoff values were then applied to each respective parameter map to generate binary output images of predicted infarct and to calculate expected infarct volume.

### Threshold-free method: Regression coefficients of perfusion parameters

For the threshold-free method, the logistic regression analysis described above was used to convert perfusion parameter values into probabilities of infarction. The model returned probability values between 0.0 and 1.0, these probabilities were directly used for infarct volume quantification without application of any thresholds. The logit model coefficients differed between patients with successful recanalization and patients with persistent occlusion. In both patient groups coefficients were consistently positive for TTD and MTT (the higher the perfusion value, the higher the probability of infarction) and negative for CBV and CBF (the lower the perfusion value, the higher the probability of infarction) (Table 3).

**Table 3:** Logistic regression coefficients of perfusion parameters.

Perfusion parameter	TICI 2b-3 (n = 93)		TICI 0-2a (n = 68)	
	Intercept	Coefficient	Intercept	Coefficient
CBF (ml × 100 g^−1^ × min^−1^)	−1.172	−0.0211	0.1188	−0.0199
CBV (ml × 100 g^−1^)	−1.323	−0.2097	−0.2044	−0.1371
MTT (s)	−2.281	0.0477	−1.137	0.0733
TTD (s)	−2.627	0.0714	−1.573	0.0987

### Method comparison: Real vs. predicted infarct volumes using the threshold-based method and the threshold-free method

**Figure 2:**
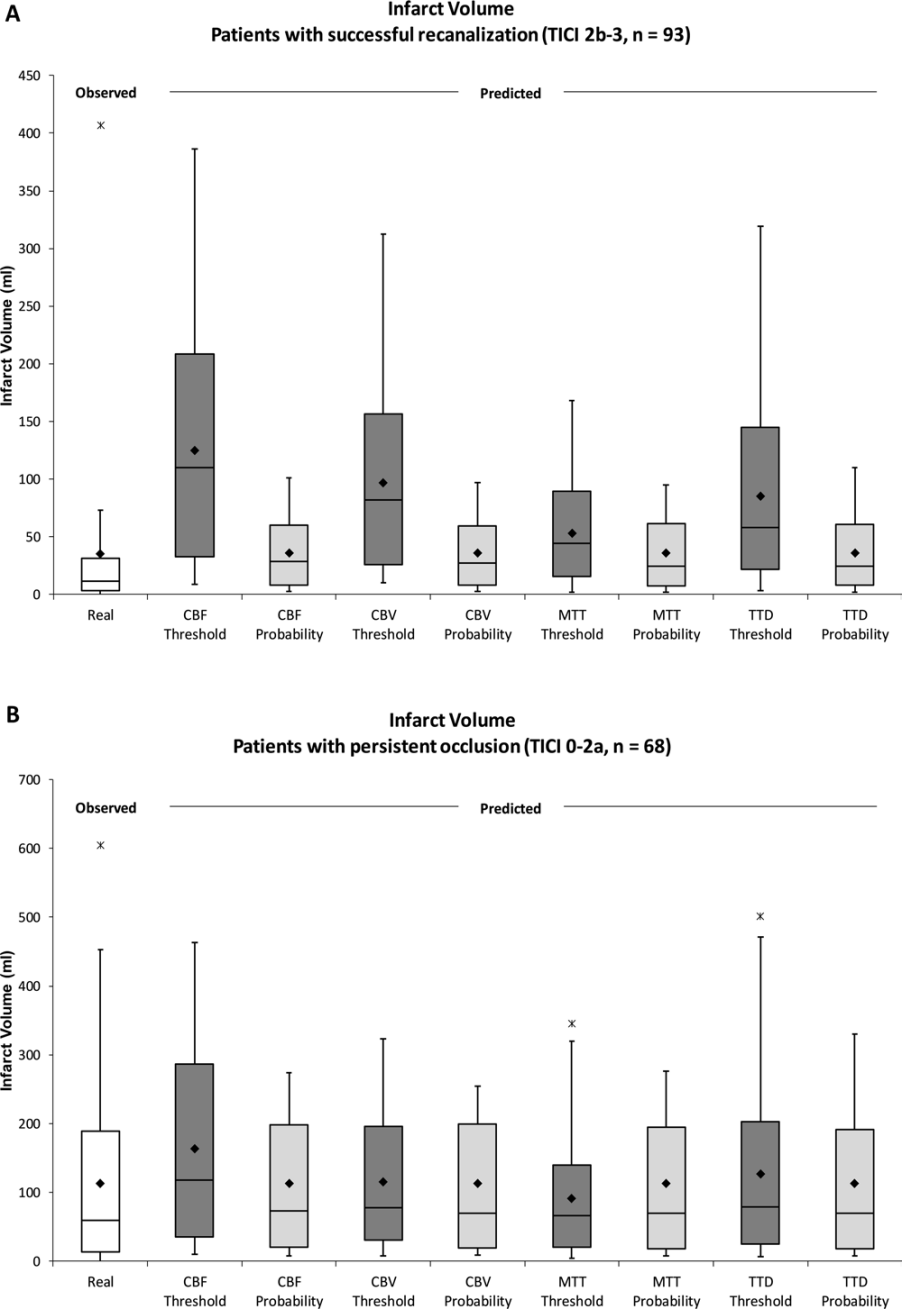
Real and predicted infarct volume for (**A**) 93 patients with successful recanalization (TICI 2b-3) and (**B**) 68 patients with persistent occlusion (TICI 0-2a). The real infarct volume and the predicted infarct volumes based on CBF, CBV, MTT, and time to drain (TTD) parameter map using the thresholding method and the probabilistic threshold-free method are shown. While the mean predicted infarct volume in the conventional threshold group does not necessarily correspond to the mean real volume, the mean predicted infarct volume predicted by the probability model fits the real mean infarct volume. ♦ = mean, horizontal line = median, x = outliers.

The real infarct volume was determined for each patient based on the manual segmentations of infarct lesions in follow-up CT datasets. Figure 2 shows the real and predicted infarct volumes for (A) 93 patients with TICI 2b-3 (successful) recanalization and (B) 68 patients with TICI 0-2a (unsuccessful) recanalization. Successfully recanalized patients showed significantly lower infarct volumes (mean (A): 35.7 ml vs. (B): 113.4 ml, p < 0.001) and smaller spread of infarct volumes (interquartile range (IQR) (A): 28.4 ml vs. (B): 175.5 ml). In both groups, smaller infarcts were more frequent than larger infarcts as box plots showed right-skewed distributions.

In patients with successful recanalization, the mean predicted infarct volume by the threshold-based method ranged from 53.2 ml (MTT) to 125.0 ml (CBF), while the IQR was lowest for MTT (74.1 ml) and highest for CBF (176.3 ml). The mean predicted infarct volume by the threshold-free method was much closer to the average real infarct volume and ranged from 35.9 ml (MTT) to 36.1 ml (TTD), while the IQR was lowest for CBV (51.7 ml) and highest for MTT (54.5 ml).

In patients with persistent occlusion, the mean predicted infarct volume by the threshold-based method ranged from 91.4 ml (MTT) to 163.8 ml (CBF), while the IQR was lowest for MTT (119.4 ml) and highest for CBF (250.8 ml). Compared to this, the mean predicted infarct volume by threshold-free analysis was again much closer to the average real infarct volume and ranged from 113.2 ml (MTT) to 113.5 ml (CBF), while the IQR was lowest for CBF (177.1 ml) and highest for CBV (179.7 ml).

### Method comparison: Prediction errors of the threshold-based method vs. threshold-free method

**Figure 3:**
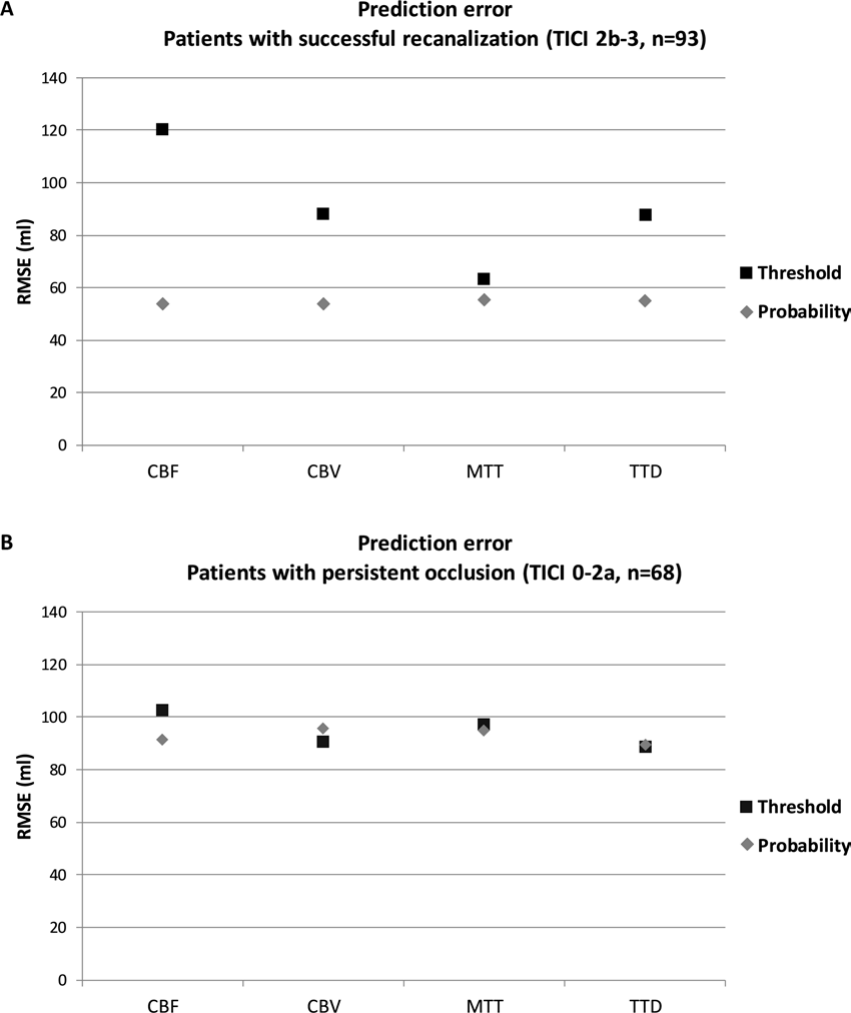
Root mean square error (RMSE) for (**A**) 93 patients with successful recanalization (TICI 2b-3) and (**B**) 68 patients with persistent occlusion (TICI 0-2a). The RMSE based on the CBF, CBV, MTT, and TTD maps using the conventional thresholding method and the probabilistic threshold-free method are shown. The prediction error is significantly lower for CBF, CBV, and TTD evaluated with the probabilistic threshold-free method compared to the threshold-based method in recanalized patients (p < 0.001).

The individual volumetric prediction error for each patient was calculated by comparing real with predicted infarct volume. Figure 3 shows the root mean square error (RMSE) for predicted volumes in (A) 93 patients with TICI 2b-3 (successful) recanalization and (B) 68 patients with TICI 0-2a (unsuccessful) recanalization.

In patients with successful recanalization, the RMSE of the threshold-based method ranged from 63.4 ml (MTT) to 120.6 ml (CBF), while the RMSE of the threshold-free method was lower ranging from 53.6 ml (CBF) to 55.4 ml (MTT). This difference of RMSE between threshold-based and threshold-free volume prediction in patients with successful recanalization was significant for CBF, CBV, and TTD (all p < 0.001). In patients with persistent occlusion, the RMSE of the threshold-based method ranged from 88.8 ml (TTD) to 102.5 ml (CBF), while the RMSE of the threshold-free method ranged from 89.6 ml (TTD) to 95.6 ml (CBV). The difference of RMSE between threshold-based and threshold-free volume prediction in patients with persistent occlusion was not statistically significant.

## Discussion

Estimating volumetric penumbra to core lesion mismatch in stroke triage has been established by prediction of infarct volumes based on univariate CT perfusion parameter analysis. CT perfusion thresholds optimized after ROC curve analysis have been traditionally used to quantify the difference between the expected infarct volume after successful vessel recanalization and permanent occlusion, the operational definition and concept of tissue at risk that may be spared from infarction if recanalization occurs.

In a well-defined multicenter population of 161 first-ever stroke patients, we compared the traditional approach of optimized thresholds of CT perfusion parameter maps for prediction of infarct volumes with a novel threshold-free probabilistic method. We report predicted infarct volumes and mean prediction error for each method when applied to four commonly used CT perfusion parameter maps in patients with successful recanalization and patients with persistent occlusion.

With respect to the volumetric estimate of the real observed brain infarcts, the threshold-free method was superior to the threshold-based method. For traditional thresholding, the optimal perfusion threshold defined by Youden-Index after ROC curve analysis did not necessarily represent the optimal threshold that predicts absolute infarct volume for individual patients. This is an important finding because tissue at risk based on optimal thresholds in perfusion parameter maps has been defined primarily in terms of abstract optimized sensitivity and specificity on a voxel level without referring to actually predicted quantitative infarct volumes. Predicted infarct volumes based on conventional optimal thresholds exhibited a substantial level of error in this study. In contrast, the probabilistic approach appeared to predict infarct volume with considerably higher accuracy and mean predicted infarct volumes were consistently closer to the real observed volumes among all patient groups and perfusion parameters. As shown by Fig. 2, both methods but especially the threshold-free approach showed a tendency towards the mean, i.e. both methods tend to overestimate small infarcts and underestimate big infarcts.

We tested four perfusion parameters (CBF, CBV, MTT and TTD) with a high reliability and reproducibility^22^. Multivariate approaches have been described in the literature, combining multiple perfusion parameters for prediction of stroke tissue outcome^13, 17, 23^. However, in the current clinical setting, thresholds are typically applied to single perfusion parameter maps to quantify volumes of infarct core and penumbra in order to select patients for therapy. Hence, to allow a direct comparison between infarct prediction by the threshold-free method and prediction by single perfusion parameter thresholds, we analyzed the four perfusion parameter maps separately in univariate analysis.

The cohort was divided into patients with successful recanalization (TICI 2b/3) and patients with persistent occlusion (TICI 0/1/2a) to differentiate between two possible therapeutic outcome scenarios: recanalization, in which infarction is typically limited to the lesion core, and persistent occlusion, in which final infarction is typically described by the lesion core and penumbra.

With regard to mean volumetric prediction error in successfully recanalized patients (n = 93), the threshold-free approach resulted in significant lower RMSE values for CBF, CBV, and TTD compared to the threshold-based method. It is important to note that follow-up infarct lesions in successfully recanalized patients have been used as reference to establish the optimal parameter that defines the lesion core at the time of admission imaging. Therefore, a reliable estimate of lesion core should particularly benefit from the threshold-free approach compared to conventional thresholding.

The spread of prediction error between the threshold-based and threshold-free approach varied among the four perfusion parameters, being highest for CBF and lowest for MTT. The significant advantage of using the threshold-free method for CBF may be explained by the fact that in critical ischemia, tissue with very low CBF values can react less favorably to recanalization compared to tissue with low CBF values^4^. A conventional perfusion parameter threshold would predict infarct in tissue for both ischemic states, while the probabilistic threshold-free approach can differentiate between low and very low values to predict volume. With regard to the more similar RMSE between the threshold-free and threshold-based method for MTT and TTD, one could hypothesize that temporal perfusion maps encoding bolus delay (such as MTT and TTD) act more like a switch, meaning that above a certain value, affected tissue will infarct no matter how fast recanalization is achieved. In this case, a probabilistic threshold-free approach will not lead to better prediction as there is no pathophysiological different risk level of infarct beyond a critical point, regardless of high versus very high values of delay.

In patients with persistent occlusion (n = 68), errors of predicted infarct volumes for the threshold-based vs. the probabilistic threshold-free approach were not significantly different. The observed significantly lower error for the probabilistic threshold-free method in successfully recanalized patients may be attributed to the overall greater variability between CT perfusion imaging and the observed tissue outcome in this group. After vessel recanalization, the extend of final infarction depends, for example, not only on the size of tissue-at-risk as defined by CTP at the time of imaging but also on the time of recanalization that occurs at some point after CTP imaging^13^. This variability has most likely a smaller effect on error for the threshold-free method than threshold-based method. In contrast, in patients with persistent occlusion, the effect of the variable time to recanalization is virtually not relevant. This may explain a more consistent relation between CT perfusion and tissue outcome in this group, and why both, the threshold-free and conventional threshold-based method, showed similar prediction errors.

In CBV and TTD maps of patients with persistent occlusion, the threshold-based approach resulted in equal or slightly lower prediction error than the probabilistic threshold-free approach (Fig. 3B, CBV and TTD in patients with persistent occlusion). This may be explained by the high variance of observed infarct volume. Because conventional thresholding makes sharp voxel-wise binary classifications (infarct, no/yes) compared to the fuzzy classification of the probabilistic approach (continuous infarct probability value), it covers a higher absolute range of infarct volume predictions (as can be seen by the higher IQR for thresholding, Fig. 2). The threshold-based predictions differed considerably from real infarct volumes in some cases (recanalized patients, CBF, Fig. 2A). Overall, threshold-free probabilistic analysis provided the best results on average with respect to volume prediction.

CT perfusion showed moderate results in terms of predictive precision reflected by AUC and volumetric error of infarct at the patient level. The AUC by ROC curve analysis for all perfusion parameters was lower than in previous publications^4, 10, 24^. First, this finding could be a result of the multi-centric origin and relatively large number of patients included in this study with less chance of overfitting compared to previously published studies establishing optimal thresholds based on much smaller and often single center cohorts. Second, inclusion criteria of large vessel occlusion could lead to a wider range of possible infarct sizes. Third, the results from ROC curve analysis depend to a large extent on the operational definition of sample space, i.e. the definition of the total brain region included for voxel analysis. The sample space may vary considerably, for example, when including all voxels belonging to the whole brain vs. ischemic hemisphere only vs. affected territory only. This definition of sample space selection, even though it is frequently omitted in past publications defining optimal thresholds, particularly affects the number of “true negatives”, i.e. the specificity with respect to observed tissue outcome. Fourth, while the average infarct outcome was predicted well across patient groups, there was a relatively high prediction error on the patient level especially in the group of recanalized patients. There seems to be crucial information missing for more precise infarct prediction after imaging in that regard. For instance, the time interval to recanalization represents a significant confounder for the benefit of recanalization that is not included in a univariate model of infarct prediction based on a single perfusion parameter map. Time intervals and other clinical variables such as age, sex, and NIHSS may be included in higher level multivariate prediction models to improve predictive power and minimize error^13^; however, this would demand efficient implementation of advanced algorithms for online results in a fast clinical routine of stroke triage which are currently not available.

There are strengths and limitations of this study. To our knowledge, this is the first study that compares predictive power of conventional perfusion thresholds to threshold free infarct prediction, and this study put both evaluated methods rigorously to the test by comparing prediction of actual infarct volumes to real outcome whereas in the past mostly results of voxel-wise ROC curve analysis without reference to actual volumes of infarct have been reported^10^.

As a limitation of patient population, the study included only endovascular treated stroke patients with large vessel occlusion. Future therapies should also include intravenously treated patients. While TICI scores are commonly dichotomized to define successful recanalization and persistent occlusion (TICI 2b/3 vs. 0/1/2a)^25, 26^, it was recently shown that stratified patients by TICI 2b and TICI 3 possibly show significant different clinical outcomes^27^. Thus, it seems convincing that both the infarct volumes, as well as optimal thresholds may change across different TICI groups.

As a limitation of image analysis, final infarction was determined on follow-up imaging 48 hours up to 7 days after stroke, which may be influenced by different degrees of brain shift due to edema. The least-square deconvolution algorithm was used to post-process raw CTP data, and results may vary for other post-processing algorithms. Optimal thresholds were determined at maximized sensitivity and specificity according to the commonly employed Youden-Index in ROC curve analysis. However, the Youden-Index assumes that incorrect classifications of infarct and no-infarct voxels are equally costly and alternative approaches to define an optimal threshold have been proposed^28^. As thresholding binarizes an image, even small changes of the cut-off value may cause significantly different volumetric predictions.

Because of technical limitations, we did not investigate the frequently used perfusion parameter time to peak of the deconvolved tissued residue function (Tmax)^29^. Although the included parameter MTT shows a good correlation with Tmax, future studies should consider Tmax for threshold-free volume prediction.

The present study focused on the precision of lesion volume prediction. Stroke volume strongly correlates with clinical outcome, and was therefore used as the main imaging endpoint in the MR-CLEAN trial^30, 31^. For both prediction methods, we treated each voxel individually where only the perfusion value per single voxel determined its tissue fate. By taking into account clustering information, i.e. the relationship between neighboring ischemic voxels, random noise artefacts could be eliminated and further improve prediction of infarct volumes. Furthermore, information of infarct clustering in specific anatomic locations may also affect prediction of clinical outcome. Because lesion pattern, besides absolute infarct volume, is an important criterion that affects severity of symptoms, secondary lesion-symptom mapping algorithms may further improve selecting patients for therapy based on expected clinical endpoints.

## Conclusions

We evaluated a probabilistic threshold-free method of CT perfusion analysis to predict infarct volume in acute ischemic stroke patients and compared this method against traditional optimal parameter thresholds in CTP imaging. The probabilistic threshold-free model predicted mean infarct volumes with higher precision and had comparable or lower patient-specific prediction errors than the threshold-based method, both in patients with successful recanalization and patients with persistent occlusion. Since infarcts after vessel recanalization vs. persistent occlusion were used to verify our model we believe these results may allow an estimate of the core to penumbra mismatch to define the tissue at risk that benefits from endovascular therapy in practice and clinical trials.

Contrary to univariate parameter thresholds, the probabilistic threshold-free method can be easily expanded to include more than one perfusion parameter in multivariate models^13^. Because predicted infarct volumes are directly calculated from the sum of continuous voxel-wise infarct probabilities, no definition of optimal thresholds is needed.
